# Dentists and the War

**Published:** 1900-01-06

**Authors:** 


### DENTISTS AND THE WAR.

MR. GORDON HOOPER, L.D.S.,[1] has drawn attention to the large amount of work for the dental surgeon which is provided by a war of any magnitude. He says that according to the report of the Surgeon-General of the American Army, 1865, from the commencement of the war to October, 1864, out of 4,167 wounds of the face there were 1,579 fractures of the facial bones, while the Crimean returns, from April 1st, 1855, to the end of the war show 533 face wounds, with 107 injuries to the bones. Under such circumstances, he inquires how far the surgeons on the spot, without special dental assistance, will be prepared to deal with bone injuries involving the jaws, and he goes on to say not only that are they not prepared to deal fully with such cases, but that the instrumental equipment necessary for so doing does not exist. There is no doubt something in all this, but, after all, the base hospitals of our South African army are surely within reach of such things. Still, it must be hard to be shut up in Ladysmith with a fractured jaw, if, as he maintains, there are not the means there for the making of proper dental splints.

[1] Journal of Tropical Medicine.

